# A graph extension of the positional Burrows–Wheeler transform and its applications

**DOI:** 10.1186/s13015-017-0109-9

**Published:** 2017-07-11

**Authors:** Adam M. Novak, Erik Garrison, Benedict Paten

**Affiliations:** 1 Genomics Institute, University of California Santa Cruz, CBSE, 501C Engineering 2, MS: CBSE, 1156 High St., Santa Cruz, CA 95064 USA; 2 Wellcome Trust Sanger Institute, Cambridge, CB10 1SA UK

**Keywords:** PBWT, Haplotype, Genome graph

## Abstract

We present a generalization of the positional Burrows–Wheeler transform, or PBWT, to genome graphs, which we call the gPBWT. A genome graph is a collapsed representation of a set of genomes described as a graph. In a genome graph, a haplotype corresponds to a restricted form of walk. The gPBWT is a compressible representation of a set of these graph-encoded haplotypes that allows for efficient subhaplotype match queries. We give efficient algorithms for gPBWT construction and query operations. As a demonstration, we use the gPBWT to quickly count the number of haplotypes consistent with random walks in a genome graph, and with the paths taken by mapped reads; results suggest that haplotype consistency information can be practically incorporated into graph-based read mappers. We estimate that with the gPBWT of the order of 100,000 diploid genomes, including all forms structural variation, could be stored and made searchable for haplotype queries using a single large compute node.

## Background

The PBWT is a compressible data structure for storing haplotypes that provides an efficient search operation for subhaplotype matches [1]. The PBWT is itself an extension of the ordinary Burrows–Wheeler transform (BWT), a method for compressing string data [2], with some concepts borrowed from the FM-index, an extension of the BWT that makes it searchable [3]. Implementations of the PBWT, such as BGT [4], can be used to compactly store and query the haplotypes of thousands of samples. The PBWT can also allow existing haplotype-based algorithms to work on much larger collections of haplotypes than would otherwise be practical [5]. The haplotype reference consortium dataset, for example, contains 64,976 haplotypes [6], and PBWT-based software allows data at this scale to efficiently inform phasing calls on newly sequenced samples, with significant speedups over other methods [7].

In the PBWT each site (corresponding to a genetic variant) is a binary feature and the sites are totally ordered. The input haplotypes to the PBWT are binary strings, with each element in the string indicating the state of a site. In the generalization we present, each input haplotype is a walk in a general bidirected graph, or genome graph. Graph-based approaches to genomics problems like mapping and variant calling have been shown to produce better results than linear-reference-based methods [8, 9], so adapting the PBWT to a graph context is expected to be useful. Other generalizations of BWT-based technologies to the graph context have been published [10–12], but they deal primarily with the substring search problem, rather than the problem of storing and querying haplotypes.

The PBWT generalization presented here allows haplotypes to be partial (they can start and end at arbitrary nodes) and to traverse arbitrary structural variation. It does not require the sites (nodes in the graph) to have a biologically relevant ordering to provide compression. However, despite these generalizations, essential features of the PBWT are preserved. The core data structures are similar, the compression still exploits genetic linkage, and the haplotype matching algorithm is essentially the same. It is expected that this generalization of the PBWT will allow large embedded haplotype panels to inform read-to-graph alignment, graph-based variant calling, and graph-based genomic data visualization, bringing the benefits of the PBWT to the world of genome graphs.

## Definitions

We define $G = (V, E)$ as a *genome graph* in a bidirected formulation [13, 14]. Each node in *V* has a DNA-sequence label; a left, or $5'$, *side*; and a right, or $3'$, side. Each edge in *E* is a pairset of sides. The graph is not a multigraph: only one edge may connect a given pair of sides and thus only one *self-loop*, or edge from a side to itself, can be present on any given side.

While more powerful algorithms are generally used in practice, a simple genome graph can be constructed relatively easily from a reference sequence and a set of nonoverlapping variants (defined as replacements of a nonempty substring of the reference with a nonempty alternate string). Start with a single node containing the entire reference sequence. For each variant to be added, break the nodes in the graph so that the reference allele of the variant is represented by a single node. Then create a node to represent the alternate allele, and attach the left and right sides of the alternate allele to everything that is attached to the left and right sides, respectively, of the reference allele.

We consider all the sides in the graph to be (arbitrarily) ordered relative to one another. We define the *null side*, 0, as a value which corresponds to no actual side in the graph, but which compares less than any actual side. We also define the idea of the *opposite* of a side *s*, with the notation $\overline{s}$, meaning the side of *s*’s node which is not *s* (i.e. the left side of the node if *s* is the right side, and the right side of the node if *s* is the left side). Finally, we use the notation *n*(*s*) to denote the node to which a side *s* belongs.

To better connect the world of bidirected graphs, in which no orientation is better than any other, and the world of algebra, in which integer subscripts are incredibly convenient, we introduce the concept of an *ambisequence*. An ambisequence is like a sequence, but the orientation in which the sequence is presented is insignificant; a sequence and its reverse are both equal and opposite *orientations* of the same underlying ambisequence. An ambisequence is isomorphic to a stick-shaped undirected graph, and the orientations can be thought of as traversals of that graph from one end to the other. For every ambisequence *s*, a canonical orientation is chosen arbitrarily, and the subscripted items $s_{i}$ are the items in that arbitrarily selected sequence. This orientation is also used for defining concepts like “previous” and “next” in the context of an ambisequence.

Within the graph *G*, we define the concept of a *thread*, which can be used to represent a haplotype or haplotype fragment. A thread *t* on *G* is a nonempty ambisequence of sides, such that for $0 \le i < N$ sides $t_{2i}$ and $t_{2i+1}$ are opposites of each other, and such that *G* contains an edge connecting every pair of sides $t_{2i}$ and $t_{2i+1}$. In other words, a thread is the ambisequence version of a walk through the sides of the graph that alternates traversing nodes and traversing edges and which starts and ends with nodes. Note that, since a thread is an ambisequence, it is impossible to reverse. Instead, the “reverse” of a thread is one of its two orientations.

We consider *G* to have associated with it a collection of *embedded* threads, denoted as *T*. We propose an efficient storage and query mechanism for *T* given *G*.

## The graph positional Burrows–Wheeler transform

Our high-level strategy is to store *T* by grouping together threads that have recently visited the same sequences of sides, and storing in one place the next sides that those threads will visit. As with the positional Burrows–Wheeler transform, used to store haplotypes against a linear reference, and the ordinary Burrows–Wheeler transform, we consider the recent history of a thread to be a strong predictor of where the thread is likely to go next [1]. By grouping together the next side data such that nearby entries are likely to share values, we can use efficient encodings (such as run-length encodings) and achieve high compression.

**Fig. 1:**
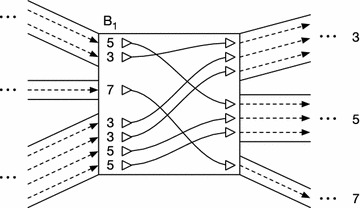
An illustration of the $B_{1}[]$ array for a single side numbered 1. (Note that a similar, reverse view could be constructed for the $B_2[]$ array and the opposite orientations of all the thread orientations shown here, but it is omitted for clarity). The* central rectangle* represents a node, and the pairs of* solid lines* on either side delimit edges attached to either the left or right side of the node, respectively. These edges connect the node to other parts of the graph, which have been elided for clarity. The* dashed lines* within the edges represent thread orientations traveling along each edge in a conserved order, while the* solid lines* with* triangles* at the ends within the displayed node represent thread orientations as they cross over one another within the node. The* triangles* themselves represent “terminals”, which connect to the corresponding* dashed lines* within the edges, and which are wired together within the node in a configuration determined by the $B_{1}[]$ array. Thread orientations entering this node by visiting side 1 may enter their next nodes on sides 3, 5, or 7, and these labels are displayed near the edges leaving the* right side* of the diagram. (Note that we are following a convention where nodes’ left sides are assigned odd numbers, and nodes’ right sides are assigned even numbers). The $B_1[]$ array records, for each thread orientation entering through side 1, the side on which it enters its next node. This determines through which of the available edges it should leave the current node. Because threads tend to be similar to each other, their orientations are likely to run in “ribbons” of multiple thread orientations that both enter and leave together. These ribbons cause the $B_s[]$ arrays to contain runs of identical values, which may be compressed.

More concretely, our approach is as follows. Within an orientation, we call an instance of side in an even-numbered position 2*i* a *visit*; a thread may visit a given side multiple times, in one or both of its orientations. (We define it this way because, while a thread contains both the left and right sides of each node it touches, we only want one visit to stand for the both of them.) Consider all visits of orientations of threads in *T* to a side *s*. For each visit, take the sequence of sides coming before this arrival at *s* in the thread and reverse it, and then sort the visits lexicographically by these (possibly empty) sequences of sides, breaking ties by an arbitrary global ordering of the threads. Then, for each visit, look two steps ahead in its thread (past *s* and $\overline{s}$) to the side representing the next visit, and append it (or the null side if there is no next visit) to a list. After repeating for all the sorted visits to *s*, take that list and produce the array $B_s[]$ for side *s*. An example *B*[] array and its interpretation are shown in Fig. 1. (Note that, throughout, arrays are indexed from 0 and can produce their lengths trivially upon demand.)

**Fig. 2:**
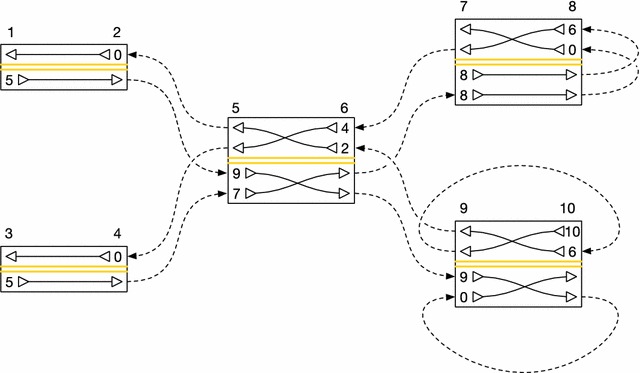
A diagram of a graph containing two embedded threads. The graph consists of nodes with sides $\{1, 2, 3, \ldots , 10\}$, connected by edges {2, 5}, {4, 5}, {6, 7}, {6, 9}, {8, 8}, and {10, 9}. Note that, once again, odd numbers are used for left sides and even numbers are used for right sides. As in Fig. 1, nodes are represented by* rectangles*, and thread orientations running from node to node are represented by* dashed lines*. The actual edges connecting the nodes are omitted for clarity; only the thread orientations are shown. Because each side’s *B*[] array defines a separate permutation, each node is divided into two parts by a central* double yellow line* (like on a road). The* top half *of each node shows visits to the node’s right side, while the* bottom half* shows visits to the node’s left side. Within the appropriate half of each node, the *B*[] array entries for the entry side are shown. The special 0 value is used to indicate that a thread stops and does not continue on to another node. When moving from the entry side to the exit side of a node, threads cross over each other so that they become sorted, stably, by the side of their next visit. Threads’ order of arrival at a node is determined by the relative order of the edges incident on the side they arrive at, which is in turn determined by the ordering of the sides on the other ends of the edges. The threads shown here are [1, 2, 5, 6, 9, 10, 9, 10] and [3, 4, 5, 6, 7, 8, 8, 7]. See Table 1 for a tabular representation of this example.

Each unoriented edge $\{ s, s' \}$ in *E* has two orientations $(s, s')$ and $(s', s)$. Let *c*() be a function of these oriented edges, such that for an oriented edge $( s, s' )$, $c(s, s')$ is the smallest index in $B_{s'}[]$ of a visit of $s'$ that arrives at $s'$ by traversing $\{ s, s' \}$. Note that, because of the global ordering of sides and the sorting rules defined for $B_{s'}[]$ above, $c(s_0, s') \le c(s_1, s')$ for $s_0 < s_1$ both adjacent to $s'$. Figure 2 and Table 1 give a worked example of a collection of *B*[] arrays and the corresponding *c*() function values.

**Table 1:** $B_s[]$ and *c*() values for the embedding of threads illustrated in Fig. 2.

Side	$B_s[]$ array
1	[5]
2	[0]
3	[5]
4	[0]
5	[9, 7]
6	[4, 2]
7	[8, 8]
8	[6, 0]
9	[9, 0]
10	[10, 6]

Edge	*c*(*s*, *t*) count
$\{2, 5\}$	0
$\{4, 5\}$	1
$\{6, 7\}$	1
$\{6, 9\}$	0
$\{8, 8\}$	0
$\{10, 9\}$	1
$\{5, 2\}$	0
$\{5, 4\}$	0
$\{7, 6\}$	0
$\{9, 6\}$	1

For a given *G* and *T*, we call the combination of the *c*() function and the *B*[] arrays a *graph positional Burrows*–*Wheeler transform (gPBWT)*. We submit that a gPBWT is sufficient to represent *T*, and, moreover, that it allows efficient counting of the number of threads in *T* that contain a given new thread as a subthread.

## Extracting threads

To reproduce *T* from *G* and the gPBWT, consider each side *s* in *G* in turn. Establish how many threads begin (or, equivalently, end) at *s* by taking the minimum of *c*(*x*, *s*) for all sides *x* adjacent to *s*. If *s* has no incident edges, take the length of $B_s[]$ instead. Call this number *b*. Then, for *i* running from 0 to *b*, exclusive, begin a new thread orientation at *n*(*s*) with the sides $[s, \overline{s}]$. Next, we traverse from *n*(*s*) to the next node. Consult the $B_s[i]$ entry. If it is the null side, stop traversing, yield the thread orientation, and start again from the original node *s* with the next *i* value less than *b*. Otherwise, traverse to side $s' = B_s[i]$. Calculate the arrival index $i'$ as $c(\overline{s}, s')$ plus the number of entries in $B_s[]$ before entry *i* that are also equal to $s'$ (i.e. the $s'$-**rank** of *i* in $B_s[]$). This arrival index, computed by the where_to function in Algorithm 1, gives the index in $B_{\overline{s'}}[]$ of the next visit in the thread orientation being extracted. Then append $s'$ and $\overline{s'}$ to the growing thread orientation, and repeat the traversal process with $i \leftarrow i'$ and $s \leftarrow s'$, until the terminating null side is reached. 

This process will enumerate both orientations of each thread in the graph. The collection of observed orientations can trivially be converted to the collection of underlying ambisequence threads *T*, accounting for the fact that *T* may contain duplicate threads. Pseudocode for thread extraction is shown in Algorithm 1. The algorithm checks each side for threads, and traces each thread one at a time, doing a constant amount of work at each step (assuming a constant maximum degree for the graph). Therefore, the algorithm runs in $O(M \cdot N + S)$ time for extracting *M* threads of length *N* from a graph with *S* sides. Beyond the space used by the gPBWT itself, the algorithm uses $O(M \cdot N)$ memory, assuming the results are stored.

This algorithm works because the thread orientations embedded in the graph run through it in “ribbons” of several thread orientations with identical local history and a conserved relative ordering. The reverse prefix sort specified in the *B*[] array definition causes thread orientations’ visits to a side *s* that come after the same sequence of immediately prior visits to co-occur in a block in $B_s[]$. For any given next side $s'$, or, equivalently, any edge $(\overline{s}, s')$, the visits to $s'$ that come after visits in that block in $B_s[]$ will again occur together and in the same relative order in a block in $B_{s'}[]$. This is because the visits at side $s'$ will share all the same history that the previous visits shared at side *s*, plus a new previous visit to *s* that no other visits to $s'$ can share. By finding a visit’s index among the visits to *s* that next take the edge from $\overline{s}$ to $s'$, and by using the *c*() function to find where in $B_{s'}[]$ the block of visits that just came from *s* starts, one can find the entry in $B_{s'}[]$ corresponding to the next visit, and thus trace out the whole thread orientation from beginning to end.

## Succinct storage

For the case of storing haplotype threads specifically, we can assume that, because of linkage, many threads in *T* are identical local haplotypes for long runs, diverging from each other only at relatively rare crossovers or mutations. Because of the reverse prefix sorting of the visits to each side, successive entries in the *B*[] arrays are thus quite likely to refer to locally identical haplotypes, and thus to contain the same value for the side to enter the next node on. Thus, the *B*[] arrays should benefit from run-length compression. Moreover, since (as will be seen below) one of the most common operations on the *B*[] arrays will be expected to be rank queries, a succinct representation, such as a collection of bit vectors or a wavelet tree [15], would be appropriate. To keep the alphabet of symbols in the *B*[] arrays small, which is beneficial for such representations, it is possible to replace the stored sides for each $B_s[]$ with numbers referring to the edges traversed to access them, out of the edges incident to $\overline{s}$.

We note that, for contemporary variant collections (e.g. the 1000 Genomes Project), the underlying graph *G* may be very large, while there may be relatively few threads (of the order of thousands) [16]. Implementers should thus consider combining multiple *B*[] arrays into a single data structure to minimize overhead.

## Embedding threads

A trivial construction algorithm for the gPBWT is to independently construct $B_s[]$ and $c(s, s')$ for all sides *s* and oriented edges $(s, s')$ according to their definitions above. However, this would be very inefficient. Here we present an efficient algorithm for gPBWT construction, in which the problem of constructing the gPBWT is reduced to the problem of embedding an additional thread.

Each thread is embedded by embedding its two orientations, one after the other. To embed a thread orientation $t = [t_0, t_1, \ldots t_{2N}, t_{2N+1}]$, we first look at node $n(t_0)$, entering by $t_0$. We insert a new entry for this visit into $B_{t_0}[]$, lengthening the array by one. The location of the new entry is near the beginning, before all the entries for visits arriving by edges, with the exact location determined by the arbitrary order imposed on thread orientations. If no other order of thread orientations suggests itself, the order created by their addition to the graph will suffice, in which case the new entry can be placed at the beginning of $B_{t_0}[]$. The addition of this entry necessitates incrementing $c(s, t_0)$ by one for all oriented edges $(s, t_0)$ incident on $t_0$ from sides *s* in *G*. We call the location of this entry *k*. The value of the entry will be $t_2$, or, if *t* is not sufficiently long, the null side, in which case we have finished the orientation.

If we have not finished the orientation, we first increment $c(s, t_2)$ by one for each side *s* adjacent to $t_2$ and after $t_1$ in the global ordering of sides. This updates the *c*() function to account for the insertion into $B_{t_2}[]$ we are about to make. We then find the index at which the next visit in *t* ought to have its entry in $B_{t_{2}}[]$, given that the entry of the current visit in *t* falls at index *k* in $B_{t_{0}}[]$. This is given by the same procedure used to calculate the arrival index when extracting threads, denoted as where_to (see Algorithm 1). Setting *k* to this value, we can then repeat the preceding steps to embed $t_2, t_3$, etc. until *t* is exhausted and its embedding terminated with a null-side entry. Pseudocode for this process is shown in Algorithm 2. 

As this algorithm proceeds, the *B*[] arrays are always maintained in the correctly sorted order, because each insertion occurs at the correct location in the array. After each *B*[] array insertion, the appropriate updates are made to the *c*() function to keep it in sync with what is actually in the array. Thus, after each thread’s insertion, the data structure correctly contains that thread, and so after the insertions of all the relevant threads, a properly constructed gPBWT is produced.

Assuming a dynamic succinct representation, where the *B*[] array information is both indexed for $O(\log (n))$ rank queries and stored in such a way as to allow $O(\log (n))$ insertion and update (in the length of the array *n*), 1 this insertion algorithm is $O(N \cdot \log (N + E))$ in the length of the thread to be inserted (*N*) and the total length of existing threads (*E*). Inserting *M* threads of length *N* will take $O(M \cdot N \cdot \log (M \cdot N))$ time, and inserting each thread will take *O*(*N*) memory in addition to the size of the gPBWT.

## Batch embedding threads

The embedding algorithm described above, Algorithm 2, requires a dynamic implementation for the succinct data structure holding the *B*[] array information, which can make it quite slow in practice due to the large constant factors involved. In order to produce a more practical implementation, it may be preferable to use a batch construction algorithm, which handles all threads together, instead of one at a time. For the case of directed acyclic graphs (DAGs), such an algorithm is presented here as Algorithm 3. 

This algorithm works essentially like the naïve trivial algorithm of independently constructing every $B_s[]$ for every side *s* and every $c(s, s')$ for every oriented edge $(s, s')$ from the definitions. However, because of the directed, acyclic structure of the graph, it is able to save redundant work on the sorting steps. Rather than sorting all the threads at each side, it sorts them where they start, and simply combines pre-sorted lists at each side to produce the *B*[] array ordering, and then stably buckets threads into new sorted lists to pass along to subsequent nodes. The directed, acyclic structure allows us to impose a full ordering on the sides in the graph, so that the sorted lists required by a side all come from “previous” sides and are always available when the side is to be processed.

Although this algorithm requires that all threads be loaded into memory at once in a difficult-to-compress representation (giving it a memory usage of $O(M \cdot N)$ on *M* threads of length *N*), and although it requires that the graph be a directed acyclic graph, it allows the *B*[] arrays to be generated for each side in order, with no need to query or insert into any of them. This means that no dynamic succinct data structure is required. Since the graph is acyclic, each thread can visit a side only once, and so the worst case is that a side is visited by every thread. Assuming a constant maximum degree for the graph, since the algorithm visits each side only once, the worst-case running time is $O(M \cdot S)$ for inserting *M* threads into a graph with *S* sides.

This algorithm produces the same gPBWT, in the form of the *B*[] arrays and the *c*() function, as the single-thread embedding algorithm would.

## Counting occurrences of subthreads

The generalized PBWT data structure presented here preserves some of the original PBWT’s efficient haplotype search properties [1]. The algorithm for counting all occurrences of a new thread orientation *t* as a subthread of the threads in *T* runs as follows.

We define $f_i$ and $g_i$ as the first and past-the-last indexes for the range of visits of orientations of threads in *T* to side $t_{2i}$, ordered as in $B_{t_{2i}}[]$.

For the first step of the algorithm, $f_0$ and $g_0$ are initialized to 0 and the length of $B_{t_0}[]$, respectively, so that they select all visits to node $n(t_0)$, seen as entering through $t_0$. On subsequent steps, $f_{i+1}$ and $g_{i+1}$, are calculated from $f_i$ and $g_i$ merely by applying the where_to function (see Algorithm 1). We calculate $f_{i+1} = \,$ where_to ${(t_{2i}, f_i)}$ and $g_{i+1} = \,$ where_to ${(t_{2i}, g_i)}$.

This process can be repeated until either $f_{i+1} \ge g_{i+1}$, in which case we can conclude that the threads in the graph have no matches to *t* in its entirety, or until $t_{2N}$, the last entry in *t*, has its range $f_N$ and $g_N$ calculated, in which case $g_N - f_N$ gives the number of occurrences of *t* as a subthread in threads in *T*. Moreover, given the final range from counting the occurrences for a thread *t*, we can count the occurrences of any longer thread that begins (in its forward orientation) with *t*, merely by continuing the algorithm with the additional entries in the longer thread.

This algorithm works because the sorting of the *B*[] array entries by their history groups entries for thread orientations with identical local histories together into contiguous blocks. On the first step, the block for just the orientations visiting the first side is selected, and on subsequent steps, the selected block is narrowed to just the orientations that visit the current side and which share the sequence of sides we have previously used in their history. The where_to function essentially traces where the first and last possible consistent thread orientations would be inserted in the next *B*[] array, and so produces the new bounds at every step.

Assuming that the *B*[] arrays have been indexed for *O*(1) rank queries (which is possible using available succinct data structure libraries such as [17], when insert operations are not required), the algorithm is *O*(*N*) in the length of the subthread *t* to be searched for, and has a runtime independent of the number of occurrences of *t*. It can be performed in a constant amount of memory (*O*(1)) in addition to that used for the gPBWT. Pseudocode is shown in Algorithm 4. 

## Results

The gPBWT was implemented within xg, the succinct graph indexing component of the vg variation graph toolkit [18]. The primary succinct self-indexed data structure used, which compressed the gPBWT’s *B*[] arrays, was a run-length-compressed wavelet tree, backed by sparse bit vectors and a Huffman-shaped wavelet tree, all provided by the sdsl-lite library used by xg [17]. The *B*[] arrays, in this implementation, were stored as small integers referring to edges leaving each node, rather than as full next-side IDs. The *c*() function was implemented using two ordinary integer vectors, one storing the number of threads starting at each side, and one storing the number of threads using each side and each oriented edge. Due to the use of sdsl-lite, and the poor constant-factor performance of dynamic alternatives, efficient integer vector insert operations into the *B*[] arrays were not possible, and so the batch construction algorithm (Algorithm 3), applicable only to directed acyclic graphs, was implemented. A modified release of vg, which can be used to replicate the results shown here, is available from https://github.com/adamnovak/vg/releases/tag/gpbwt2.

The modified vg was used to create a genome graph for human chromosome 22, using the 1000 Genomes Phase 3 VCF on the GRCh37 assembly, embedding information about the correspondence between VCF variants and graph elements [16]. Note that the graph constructed from the VCF was directed and acyclic; it described only substitutions and indels, with no structural variants, and thus was amenable to the batch gPBWT construction algorithm. Next, haplotype information for the 5008 haplotypes stored in the VCF was imported and stored in a gPBWT-enabled xg index for the graph, using the batch construction algorithm mentioned above. In some cases, the VCF could not be directly translated into self-consistent haplotypes. For example, a G to C SNP and a G to GAT insertion might be called at the same position, and a haplotype might claim to contain the alt alleles of both variants. A naïve interpretation might have the haplotype visit the C and then the GAT, which would be invalid, because the graph would not contain the C to G edge. In cases like this, an attempt was made to semantically reconcile the variants automatically (in this case, as a C followed by an AT), but this was only possible for some cases. In other cases, invalid candidate haplotype threads were still generated. These were then split into valid pieces to be inserted into the gPBWT. Threads were also split to handle other exceptional cases, such as haploid calls in the input. Overall, splitting for causes other than loss of phasing occurred 203,145 times across the 5008 haplotypes, or about 41 times per haplotype.

The xg indexing and gPBWT construction process took 9 h and 19 min using a single indexing thread on an Intel Xeon X7560 running at 2.27 GHz, and consumed 278 GB of memory. The high memory usage was a result of the decision to retain the entire data set in memory in an uncompressed format during construction. However, the resulting xg index was 436 MB on disk, of which 321 MB was used by the gPBWT. Information on the 5008 haplotypes across the 1,103,547 variants was thus stored in about 0.93 bits per diploid genotype in the succinct self-indexed representation, or 0.010 bits per haplotype base.^2^ Extrapolating linearly from the 51 megabases of chromosome 22 to the entire 3.2 gigabase human reference genome, a similar index of the entire 1000 Genomes dataset would take 27 GB, with 20 GB devoted to the gPBWT. This is well within the storage and memory capacities of modern computer systems.

### Random walks

**Fig. 3:**
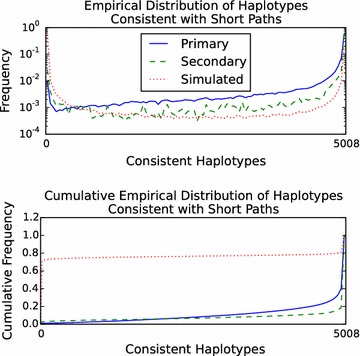
Distribution (*top*) and cumulative distribution (*bottom*) of the number of 1000 Genomes Phase 3 haplotypes consistent with short paths in the GRCh37 chromosome 22 graph. Primary mappings of 101 bp reads with scores of 90 out of 101 or above ($n=1,500,271$) are the* solid blue line*. Secondary mappings meeting the same score criteria ($n=43,791$) are the* dashed green line*. Simulated 100 bp random walks in the graph without consecutive N characters ($n=686,590$) are the* dotted red line*. Consistent haplotypes were counted using the gPBWT support added to vg [18].

The query performance of the gPBWT implementation was evaluated using random walk query paths. 1 million random walks of 100 bp each were simulated from the graph. To remove walks covering ambiguous regions, walks that contained two or more N bases in a row were eliminated, leaving 686,590 random walks. The number of haplotypes in the gPBWT index consistent with each walk was then determined, taking 61.29 s in total using a single query thread on the above-mentioned Xeon system. The entire operation took a maximum of 458 MB of memory, indicating that the on-disk index did not require significant expansion during loading to be usable. Overall, the gPBWT index required 89.3 μs per count operation on the 100 bp random walks. It was found that 316,078 walks, or 46%, were not consistent with any haplotype in the graph. The distribution of of the number of haplotypes consistent with each random walk is visible in Fig. 3.

#### Read alignments

To further evaluate the performance of the query implementation, we evaluated read alignments to measure their consistency with stored haplotypes. 1000 Genomes Low Coverage Phase 3 reads for NA12878 that were mapped in the official alignment to chromosome 22 were downloaded and re-mapped to the chromosome 22 graph, using the xg/GCSA2-based mapper in vg, allowing for up to a single secondary mapping per read. (The vg aligner was chosen because of its ease of integration with our xg-based gPBWT implementation, but in principle any aligner that supports aligning to a graph could be used.) The mappings with scores of at least 90 points out of a maximum of 101 points (for a perfectly-mapped 101 bp read) were selected (thus filtering out alignments highly like to be erroneous) and broken down into primary and secondary mappings. The number of haplotypes in the gPBWT index consistent with each read’s path through the graph was calculated (Fig. 3). For 1,500,271 primary mappings, the count operation took 150.49 seconds in total, or 100 microseconds per mapping, using 461 MB of memory. (Note that any approach that depended on visiting each haplotype in turn, such as aligning each read to each haplotype, would have to do its work for each read/haplotype combination in less than 20 μs, or about 45 clock cycles, in order to beat this time.) It was found that 2521 of these primary mappings, or 0.17%, and 320 of 43,791 secondary mappings, or 0.73%, were not consistent with any haplotype path in the graph.^3^ These read mappings, despite having reasonable edit based scores, may represent rare recombinations, but the set is also likely to be enriched for spurious mappings.

### Scaling characteristics

**Fig. 4:**
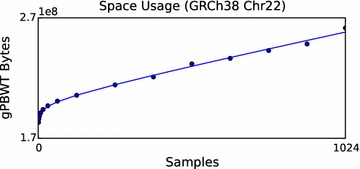
Disk space usage for the gPBWT versus sample count for GRCh38 chromosome 22. Points are sampled at powers of two up to 128, and intervals of 128 thereafter up to 1024. The trend line shown corresponds to the function $y(x) = {3.16}\times 10^{6} \mathrm {bytes} \cdot \ln (x / \mathrm {samples}) + {5.12}\times 10^{4}\frac{\mathrm {bytes}}{\mathrm {sample}} \cdot x + {1.84}\times 10^{8}\mathrm {bytes}$.

To evaluate the empirical space usage scaling characteristics of our gPBWT implementation, a scaling experiment was undertaken. The 1000 Genomes Phase 3 VCFs for the GRCh38 assembly were downloaded, modified to express all variants on the forward strand in the GRCh38 assembly, and used together with the assembly data to produce a graph for chromosome 22 based on the newer assembly. This graph was then used to construct a gPBWT with progressively larger subsets of the available samples. Samples were selected in the order they appear in the VCF file. For each subset, an xg serialization report was generated using the xg tool, and the number of bytes attributed to “threads” was recorded. The number of bytes used versus the number of samples stored is displayed in Fig. 4.

After empirical size data was obtained, a log-plus-linear curve, consisting of a log component and a linear component, was fit to the data. This curve was used to extrapolate an estimated size of 5.34 GB on disk for the storage of 100,000 samples’ worth of data on chromosome 22. We choose 100,000 because it is representative of the scale of large contemporary sequencing projects, such as Genomics England’s 100,000 Genomes Project (https://www.genomicsengland.co.uk/the-100000-genomes-project/) [20] and the NHLBI’s TOPMed program (https://www.nhlbi.nih.gov/research/resources/nhlbi-precision-medicine-initiative/topmed). Linear extrapolation from the 51 megabase chromosome 22 to the 3.2 gigabase human genome yields a size estimate of 336 GB for the storage of 100,000 diploid genomes, in addition to the space usage of the underlying graph. Although this extrapolation does not account for the dependence of graph complexity on the number of samples sequenced, it suggests that the gPBWT is capable of scaling to the anticipated size of future sequencing data sets, while using currently available computing resources.

## Discussion

We have introduced the gPBWT, a graph based generalization of the PBWT. We have demonstrated that a gPBWT can be built for a substantial genome graph (all of human chromosome 22 and the associated chromosome 22 substitutions and indels in 1000 Genomes). Using this data structure, we have been able to quickly determine that the haplotype consistency rates of random walks and primary and secondary read mappings differ substantially from each other, and based on the observed distributions we hypothesize that consistency with very few haplotypes can be a symptom of a poor alignment.

Such poor alignments could arise by a variety of means, including similarity between low complexity sequence, or paralogy, the latter representing true sequence homology but not true sequence orthology. Paralogous alignments are often difficult to distinguish from truly orthologous alignments, and can lead to the reporting of false or misplaced variants. Using haplotype consistency information is one way we might better detect paralogy, because paralogous sequence is not expected to be consistent with linkage relationships at a paralogous site. A more sophisticated analysis of haplotype consistency rate distributions could thus improve alignment scoring.

In the present experiment, we have examined only relatively simple variation: substitutions and short indels. Instances of more complex variation, like large inversions and translocations, which would have induced cycles in our genome graphs, were both absent from the 1000 Genomes data set we used and unsupported by the optimized DAG-based construction algorithm which we implemented. We expect that complex structural variation is well suited to representation as a genome graph, so supporting it efficiently should be a priority for a serious practical gPBWT construction implementation.

Extrapolating from our results on chromosome 22, we predict that a whole-genome gPBWT could be constructed for all 5008 haplotypes of the 1000 Genomes data on GRCh37 and stored in the main memory of a reasonably apportioned computer, using about 27 GB of memory for the final product. On the GRCh38 data set, we extrapolate a space usage of 21 GB for the 2504 samples of the 1000 Genomes Project; a whole-genome gPBWT for 100,000 samples on GRCh38, we predict, could be stored in about 336 GB. Computers with this amount of memory, though expensive, are readily available from major cloud providers. (The wasteful all-threads-in-memory construction implementation we present here, however, would not be practical at such a scale, requiring on the order of 50 TB of memory to handle 100,000 samples when constructing chromosome 1; a disk-backed implementation or other low-memory construction algorithm would be required.) The relatively modest growth from 5008 haplotypes (2504 samples) to 200,000 haplotypes (100,000 samples) is mostly attributable to the run-length compression used to store the *B* arrays in our implementation. Each additional sample is representable as a mere increase in run lengths where it agrees with previous samples, and contributes an exponentially diminishing number of new variants and novel linkage patterns. While further empirical experimentation will be necessary to reasonably extrapolate further, it does not escape our notice that the observed scaling patterns imply the practicality of storing cohorts of a million or more individuals, such as those envisaged by the Precision Medicine Initiative [21] and other similar national efforts, within an individual powerful computer. Looking forward, this combination of genome graph and gPBWT could potentially enable efficient mapping not just to one reference genome or collapsed genome graph, but simultaneously to an extremely large set of genomes related by a genome graph.

## Abbreviations

BWT: Burrows–Wheeler transform
PBWT: positional Burrows–Wheeler transform
gPBWT: graph positional Burrows–Wheeler transform
GRC: genome reference consortium
GRCh37: GRC human genome assembly, build 37
GRCh38: GRC human genome assembly, build 38
DAG: directed acyclic graph

## Footnotes

1 Dynamic data structures at least this good are available as part of the DYNAMIC library, from https://github.com/xxsds/DYNAMIC.

2 The improved size results here relative to the results in our conference paper are related to the use of a new run-length-compressed storage backend for the *B*[] arrays, replacing one that was previously merely succinct [19].

3 These numbers are expected to differ from those reported in our conference paper due to improvements to the vg mapping algorithms since the conference paper was prepared [19].
